# Diversity, function and assembly of mangrove root-associated microbial communities at a continuous fine-scale

**DOI:** 10.1038/s41522-020-00164-6

**Published:** 2020-11-12

**Authors:** Wei Zhuang, Xiaoli Yu, Ruiwen Hu, Zhiwen Luo, Xingyu Liu, Xiafei Zheng, Fanshu Xiao, Yisheng Peng, Qiang He, Yun Tian, Tony Yang, Shanquan Wang, Longfei Shu, Qingyun Yan, Cheng Wang, Zhili He

**Affiliations:** 1 Environmental Microbiomics Research Center, School of Environmental Science and Engineering, Southern Marine Science and Engineering Guangdong Laboratory (Zhuhai), South China Sea Institution, Sun Yat-sen University, 510006 Guangzhou, China; 2 Department of Civil and Environmental Engineering, The University of Tennessee, Knoxville, TN 37996 USA; 3 Key Laboratory of the Ministry of Education for Coastal and Wetland Ecosystems, School of Life Sciences, Xiamen University, 361102 Xiamen, China; 4 Swift Current Research and Development Centre, Agriculture and Agri-Food Canada, Swift Current, SK S9H 3×2 Canada; 5 College of Agronomy, Hunan Agricultural University, 410128 Changsha, China

**Keywords:** Microbial ecology, Environmental microbiology

## Abstract

Mangrove roots harbor a repertoire of microbial taxa that contribute to important ecological functions in mangrove ecosystems. However, the diversity, function, and assembly of mangrove root-associated microbial communities along a continuous fine-scale niche remain elusive. Here, we applied amplicon and metagenome sequencing to investigate the bacterial and fungal communities among four compartments (nonrhizosphere, rhizosphere, episphere, and endosphere) of mangrove roots. We found different distribution patterns for both bacterial and fungal communities in all four root compartments, which could be largely due to niche differentiation along the root compartments and exudation effects of mangrove roots. The functional pattern for bacterial and fungal communities was also divergent within the compartments. The endosphere harbored more genes involved in carbohydrate metabolism, lipid transport, and methane production, and fewer genes were found to be involved in sulfur reduction compared to other compartments. The dynamics of root-associated microbial communities revealed that 56–74% of endosphere bacterial taxa were derived from nonrhizosphere, whereas no fungal OTUs of nonrhizosphere were detected in the endosphere. This indicates that roots may play a more strictly selective role in the assembly of the fungal community compared to the endosphere bacterial community, which is consistent with the projections established in an amplification-selection model. This study reveals the divergence in the diversity and function of root-associated microbial communities along a continuous fine-scale niche, thereby highlighting a strictly selective role of soil-root interfaces in shaping the fungal community structure in the mangrove root systems.

## Introduction

Mangroves account for 60–70% of tropical and sub-tropical coastlines worldwide^1^ and have tremendous ecological importance as they participate in elemental cycling^2–4^, mediate global climate change^3^, protect coastlines^5^, and facilitate phytoremediation^1^. Similar to typical terrestrial plants, mangroves depend upon mutually beneficial interactions with microbial communities^1^. In particular, microbes residing in developed roots could help mangroves transform nutrients into usable forms prior to plant assimilation^2,6^. These microbes also provide mangroves phytohormones for suppressing phytopathogens^7^ or helping mangroves withstand heat and salinity^1^. In turn, root-associated microbes receive carbon metabolites from the plant via root exudates^8^, thus close associations between the plant and microbes are established for their mutual benefits^9^.

Highly diverse microbial communities (mainly bacteria and fungi) have been found to inhabit and function in mangrove roots^5,10,11^. For example, diazotrophic bacteria in the vicinity of mangrove roots could perform biological nitrogen fixation, which provides 40–60% of the total nitrogen required by mangroves^12,13^; the soil attached to mangrove roots lacks oxygen but is rich in organic matter, providing an optimal microenvironment for sulfate-reducing bacteria (SRB) and methanogens^1^; ligninolytic, cellulolytic, and amylolytic fungi are prevalent in the mangrove root environment^10^; rhizosphere fungi could help mangroves survive in waterlogged and nutrient-restricted environments^14^. These studies have provided increasing evidences to support the importance of root-associated bacteria and fungi for mangrove growth and health^1,2^. However, systematic field studies on the overall taxonomic and functional diversity of mangrove root-associated microbial communities are still limited beyond examples of specific types of functional microbial members^10,12–14^.

Recent studies have investigated the detailed structure of root-associated microbial communities at a continuous fine-scale in other plants^15^, where a microhabitat was divided into four root compartments: endosphere^7,15,16^, episphere^7^, rhizosphere^15,17^, and nonrhizosphere^18,19^. Moreover, the microbial communities in each compartment have been reported to have unique characteristics^7,15^. The rhizosphere could emit root exudates that selectively enriched specific microbial populations; however, these exudates were found to exert only marginal impacts on microbes in the nonrhizosphere soil^8,9^. Furthermore, it was noted that the root episphere, rather than the rhizosphere, was primarily responsible for controlling the entry of specific microbial populations into the root^7^, resulting in the selective enrichment of Proteobacteria in the endosphere^7,20^. These findings provide new insights into the niche differentiation of root-associated microbial communities^7–9,20^. Nevertheless, amplicon-based community profiling may not provide the functional characteristics of root-associated microbial communities in plant growth and biogeochemical cycling^21^. Unraveling functional patterns across the four root compartments holds a great potential for understanding functional mechanisms responsible for mediating root–microbe interactions in support of enhancing mangrove ecosystem functioning.

Recently, root exudates were reported to be well-known determinants of root-associated microbial assemblages. Root exudation is a spatially defined process that contributes to distinct microbial communities that have been linked to specific root compartments^8^. This is partly because root exudates could act as a carbon sources and alter the rhizosphere pH^7,8,15^. However, the effect of root exudates on rhizobiome assembly is complicated, and the strategy of root-associated microbial community assembly at the soil-root interface remains controversial. The findings of some studies on root-associated microbiota in rice corroborate a two-step or multiple-step model in the root microbiota assembly, where specific microbial taxa in soil gradually become depleted or enriched during root colonization^15,22^. Other studies on rice and Medicago root-associated microbiota pointed to the applicability of the amplification-selection process, where dominant phyla would undergo substantial enrichment in the rhizosphere followed by the specific recruitment of certain phyla into the roots^23^. We aimed to determine the assembly processes that shape mangrove root-associated microbiota and examine the extent of differentiation and enrichment of mangrove root-specific microbial taxa across four root compartments. Furthermore, a community assembly framework developed by Vellend^24^ and modified by Stegen et al.^25^ allowed us to disentangle the ecological processes (heterogeneous selection, homogeneous selection, homogeneous dispersal, dispersal limitation, and undominated processes) that drive the mangrove root-associated microbial community composition at the spatial scale.

In this study, we used 16S rRNA and internal transcribed spacer (ITS) gene amplicon and metagenomic sequencing to systematically explore the diversity, function, and assembly of root-associated microbial communities in four continuous fine-scale root compartments (nonrhizosphere, rhizosphere, episphere, and endosphere) of *Kandelia obovata* (KO), a native mangrove plant of Southern China^26^. We hypothesized that the diversity and function of mangrove root-associated microbial communities at the fine-scale could be largely affected by niche differentiation along the root compartments, and that the secretion of root exudates would affect root microbiomes in microbe-soil-plant systems^15^. This study provides new insights into the understanding of root-associated microbial communities and their assembly mechanisms in mangrove ecosystems.

## Results

### Diversity and composition of microbial communities among four mangrove root compartments

**Fig. 1:**
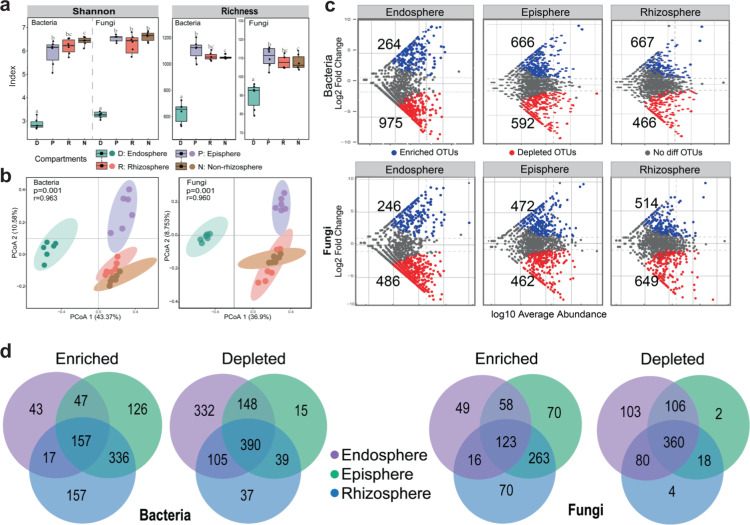
Microbial diversity and enriched/depleted OTUs among four mangrove root compartments. **a** Boxplot of Shannon index and richness. The horizontal line that intersects the box is the median. The tops and bottoms of boxes represent 75th and 25th quartiles, respectively. The upper and lower whiskers extend 1.5× the interquartile range from the upper edge and lower edge of the box, respectively. Letters depict significant differences across compartments. **b** Principal coordinates analysis (PCoA) of bacterial and fungal communities, and the 95% confidence ellipses are shown around the samples and grouped based on four root-related compartments. **c** Enriched and depleted bacterial and fungal OTUs in rhizosphere, episphere, and endosphere compartments compared to the non-rhizosphere control. Each point represents an individual OTU, and the position along the *y*-axis represents the abundance fold change. **d** Number of differentially enriched and depleted OTUs of bacteria and fungi in each compartment. N: non-rhizosphere, R: rhizosphere, P: episphere, D: endosphere.

To determine whether microbial diversity varied across four continuous fine-scale compartments, we analyzed mangrove root-associated bacterial and fungal communities by sequencing 16S rRNA and ITS gene amplicons. Our amplicon sequencing analysis revealed a substantial difference in the diversity of bacterial and fungal communities among the compartments of nonrhizosphere (N), rhizosphere (R), episphere (P), and endosphere (D) (Fig. 1a). We observed the lowest Shannon-diversity and operational taxonomic unit (OTU) richness in the endosphere for both bacterial and fungal communities. The non-rhizosphere exhibited a higher bacterial diversity than the episphere, although fungal diversity indices were similar among the three exterior compartments (N, R, and P). Principal coordinate analysis (PCoA) based on Bray–Curtis distances showed that bacterial and fungal communities in the four root compartments were well-separated (*P* < 0.05, Adonis test), with the endosphere samples distinctively separated from the samples in other compartments (Fig. 1b).

The detected OTUs were distributed across seven dominant phyla. The abundance of Proteobacteria increased gradually from the non-rhizosphere to the endosphere, whereas the abundance of Chloroflexi decreased (Supplementary Fig. 1). As the most distinctive compartment, the endosphere was dominated by Proteobacteria (69.83%), and contained a low abundance of Chloroflexi (7.93%) and Actinobacteria (4.24%) (Supplementary Fig. 1a). The enrichment of a highly diverse Proteobacteria community in the endosphere was accompanied by the dominance of the following bacterial families (Supplementary Fig. 1b): two Alpha-proteobacterial families (Hyphomicrobiaceae and Rhodobacteraceae, 11.25% and 5.24%, respectively), two Gamma-proteobacterial families (Vibrionaceae and Saccharospirillaceae, 11.48% and 2.94%, respectively), and one Delta-proteobacterial family (Desulfobulbaceae, 10.72%). At the genus level, we also observed notable differences among the four root compartments. The endosphere had a significantly greater proportion of *Vibrio* and *Saccharospirillum* than other compartments (*P* < 0.05, Student’s *t*-test), whereas *Desulfococcus*, *Desulfosarcina*, and *Defluviitalea* were mostly depleted in the endosphere compared to the other three compartments (Supplementary Fig. 1c). In line with bacterial communities, fungal communities also showed significant variations across the four root compartments (*P* < 0.05, Student’s *t*-test). As the two dominant known fungal phyla in the four root compartments, Ascomycota (11%) and Basidiomycota (17%) in the endosphere had a lower abundance than those in other compartments (Ascomycota: 26%; Basidiomycota: 22%) (Supplementary Fig. 2). This variation trend across the four root compartments was also apparent in the abundant OTUs belonging to the unclassified fungi (Supplementary Fig. 2).

### Enriched or depleted microbial OTUs among four mangrove root compartments

To identify the OTUs that contributed to the divergence in microbial community composition in the four root compartments, we conducted differential abundance analyses using a negative binomial distribution with OTU counts. Non-rhizosphere soils were set as the control and 0.01 as the adjusted *P* value cutoff. From the exterior (rhizosphere) to interior (endosphere) root compartments, the number of detected OTUs in bacterial communities was similar (R-P-D: 1133-1258-1239), but that in fungal communities showed a decreasing trend (R-P-D: 1163-934-732). The numbers of the enriched bacterial OTUs (R-P-D: 466-592-975) and the depleted fungal OTUs (R-P-D: 514-472-246) exhibited a completely different trend. Such differences were notable in the endosphere, which harbored more depleted bacterial OTUs (975) but less enriched bacterial OTUs (264) and fungal OTUs (246) compared with those in the episphere and rhizosphere (Fig. 1c).

We also observed noteworthy overlaps and distinctions in the abundant OTUs in the four root compartments. We found that 73.9% and 26.1% of rhizosphere-enriched bacterial OTUs were enriched in the episphere and endopshere, respectively. Likewise, 53.7% and 27.0% of rhizosphere-enriched fungal OTUs were also enriched in the episphere and endopshere, respectively. These data suggest that many microbes in the rhizosphere were able to colonize the root. Apart from these overlaps, considerable distinct OTUs were observed in each root compartment, as almost all of the depleted OTUs in the rhizosphere were depleted in the episphere, endosphere or both. For example, 55.2% of bacterial OTUs and 77.8% of fungal OTUs depleted in the episphere were also significantly depleted in the endosphere (*P* < 0.05) (Fig. 1d). Collectively, root-associated bacterial and fungal communities formed four spatially separable root compartments with distinct and overlapping microbial taxa.

### Functions of root-associated microbial communities in the four mangrove root compartments

**Fig. 2:**
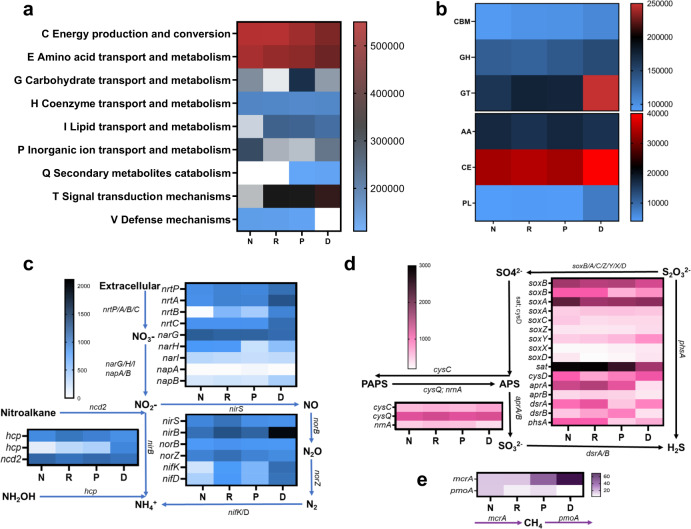
Shotgun metagenome sequencing analysis of four mangrove root-compartments microbiota. Heatmaps depict the relative abundances of **a** EggNOG modules, **b** CAZy modules, and key genes involved in **c** nitrogen cycling, **d** sulfur cycling, and **e** methanogenesis.

To explore microbial functions in the four root compartments, we analyzed mangrove root-associated microbial communities using shotgun metagenome sequencing with a focus on the relative abundance of key functional genes and pathways involved in carbon, nitrogen, sulfur and methane cycling (Fig. 2). The metagenomic contigs were annotated using evolutionary genealogy of genes: Non-supervised Orthologous Groups (eggNOG), Carbohydrate-Active enZymes (CAZy), and Kyoto Encyclopedia of Genes and Genomes (KEGG) databases. We found that the niche differentiation of root-associated microbial communities was accompanied by a notable divergence of microbial functions among the four root compartments (Fig. 2).

First, the functional annotations by eggNOG process categories indicated that the genes involved in carbohydrate transport and metabolism (G) were more abundant in the endosphere than in the rhizosphere and episphere (Fig. 2a). Also, the genes involved in secondary metabolite biosynthesis, transport, and catabolism (Q) were enriched in the endosphere and episphere, while they were depleted in the rhizosphere and non-rhizosphere. The endosphere, episphere, and rhizosphere consisted of many genes for lipid transport and metabolism (I) and signal transduction mechanisms (T). Through the analysis of CAZy reference sequences that are closely related to carbon cycling (Fig. 2b), we observed that the endosphere had the highest abundance of six CAZy families, with the enrichment of carbohydrate esterase (CE), glucosyltransferase (GT), and glycoside hydrolase (GH) genes (Fig. 2b).

Second, as nitrogen limitation of mangrove sediments can restrict plant growth and microbial activity, we estimated the abundance of a variety of functional genes involved in nitrogen cycling in the mangrove root environment (Fig. 2c). We found an increase in the relative abundance of genes involved in the conversion of extracellular polymers into NO_3_^−^ (*nrtA/B/C*) from the outside to the inside of root [gene transcripts per million (TPM) values in N and D were 1572 and 2986, respectively]. Although genes that allow the conversion of NO_2_^−^, NH_2_OH, and N_2_ (*nirB, hcp*, and *nifK*, respectively) were enriched in the four root compartments (Fig. 2c), the endosphere had the highest abundance of *nifK* (934) and *nirB* (2119) and a lower abundance of *nirS* compared to other compartments (Fig. 2c).

Also, for sulfur cycling genes, the average abundance of *soxB/A/C/Z/Y/X/D* in soil (7202) was higher than that in the rhizosphere, episphere, and endosphere (R-P-D: 6702-6623-6651), indicating that the potential of S_2_O_3_^−^ conversion to SO_4_^2−^ decreased around the root. Functional genes, including *sat*, *cysD*, *phsA*, and *dsrB*, showed the lowest abundance in the endosphere, whereas *dsrA* was of minimum abundance in the episphere (Fig. 2d). These data indicate that sulfur reduction potential may be greater in the non-rhizosphere and rhizosphere soils.

In addition, mangrove ecosystems have been proposed as important methane sinks^3^, thus we estimated the abundance of functional genes related to methanogenesis and methanotrophy (Fig. 2e). Our data indicated that the abundance of methyl coenzyme M reductase gene (*mcrA)* in the non-rhizosphere (17) was lower than that in the endosphere (69), where the particulate methane monooxygenase (*pmoA)* was of the lowest abundance (Fig. 2e). The highest ratio of *mcrA to pmoA* (27) was observed in the endosphere, suggesting that mangrove roots have a high potential for CH_4_ production. Altogether, the above results revealed that root-associated microbial communities could play an important role in carbon, nitrogen, and sulfur cycling. More importantly, metabolic functional potentials differed substantially among the four mangrove root compartments.

### Root exudates could shape mangrove root-associated microbial communities

**Fig. 3:**
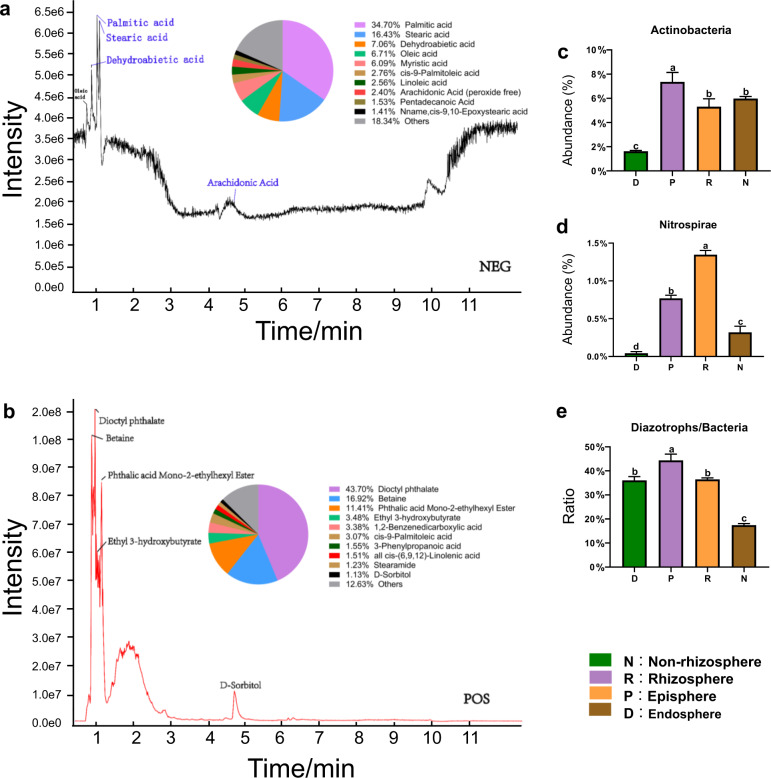
Metabolite analysis of *K. obovata* root exudation. Untargeted metabolomic analysis of root exudates with **a** POS and **b** NEG model. The pie chart shows the relative abundance of top ten exudate compounds in mangrove roots. The relative abundance of **c** Actinobacteria and **d** Nitrospirae among four root compartments were determined via amplicon sequencing. **e** The ratios of diazotrophs to bacteria among four mangrove root compartments were determined using qPCR. Error bars correspond to the standard deviation (s.d.).

To test whether root exudates regulate the diversity and composition of mangrove root-associated microbial communities, we measured the components of *K. obovata* root exudates using untargeted metabolomic analysis with negative mode acquisition (NEG) and positive mode acquisition (POS) modules. We identified 216 metabolites in mangrove root exudates, including amino acids, organic acids, polyhydroxy acids, sugars, phosphates, polyols, and N-compounds (Fig. 3a). The most abundant metabolites identified in the NEG module included palmitic acid (34.7%), stearic acid (16.4%), dehydroabietic acid (7.1%), oleic acid (6.7%), and myristic acid (6.1%), and those identified in POS module were dioctyl phthalate (43.7%), betaine (16.9%), phthalic acid mono-2-ethylhexyl ester (11.4%), and ethyl 3-hydroxybutyrate (3.5%).

Actinobacteria, Nitrospirae (diazotrophs), and mycorrhizal fungi, as members of root-associated microbial communities, have been reportedly to be correlated with some fatty acids (palmitic, linoleic, oleic, and stearic)^8^; we, therefore, compared the relative abundance of these populations among the four root compartments. Our data showed that the relative abundance of Actinobacteria (7.4%) was prominently higher in the episphere than in other compartments (Fig. 3c), which corroborates the finding that Actinobacterial members were the major groups stimulated by plant root exudates^27^. Likewise, diazotrophs were also specifically enriched in the rhizosphere and episphere (Fig. 3d, e). Other microbial taxa associated with root exudates^8,9^ such as Firmicutes, Chloroflex, and Ascomycota, were also significantly enriched (*P* < 0.05) in the episphere (Fig. 1e). Considering non-rhizosphere as the control, we found that *Hyphomicrobium* in the episphere and rhizosphere was positively correlated with a variety of abundant constituents of root exudates, including betaine, salicylic acid, myristic acid, oleic acid, stearic acid, and palmitic acid (Supplementary Fig. 3). Therefore, we proposed that root exudates in mangroves could profoundly influence many root-associated microbial populations, especially those inhabiting the episphere.

### Assembly mechanisms for microbial communities in mangrove root compartments

**Fig. 4:**
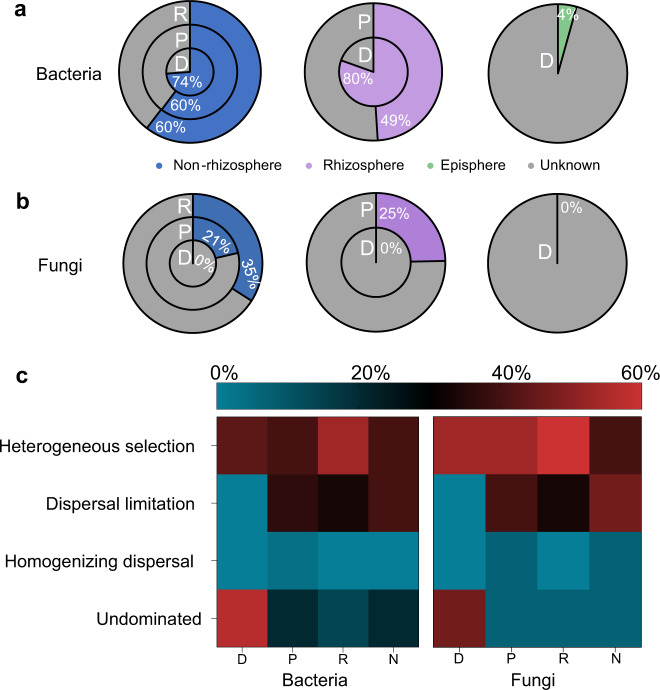
Microbial acquisition along the soil-root continuum. SourceTracker results are shown to estimate the probability of **a** bacteria and **b** fungi derived from the exterior compartments. Non-rhizosphere, rhizosphere, and episphere were chosen as the individual source, and their corresponding results are illustrated in the left, middle, and right circle, respectively. **c** The ecological processes of bacteria and fungi among four mangrove root compartments.

To understand the dynamics of mangrove root-associated microbial communities, we combined the use of source-tracker and ecological process analyses to elucidate microbial acquisition along the soil-root continuum. The results revealed that most bacterial OTUs in the endosphere were derived from the non-rhizosphere (74%), and only a small part of the episphere bacterial communities (4%) were detected in the endosphere compartment (Fig. 4a). Moreover, no fungal OTUs that were previously detected in the non-rhizosphere or episphere were detected in the endosphere (Fig. 4b). To verify the dissimilar manner of acquisition of bacteria and fungi by the root, we used real-time quantitative PCR (qPCR) to determine the ratio of bacteria to fungi among the four root compartments (Supplementary Fig. 4). The results showed that the ratio of bacteria to fungi decreased from 128.0% in the non-rhizosphere to 13.8% in the endosphere. Furthermore, ecological process analysis revealed that the deterministic processes of heterogeneous selection contributed >40% to the community assembly for both bacteria and fungi (Fig. 4c). Altogether, the results suggested that the episphere could effectively act as a gate for controlling the entry of microbes into the root endosphere, revealing a dominance of heterogeneous selection in the assembly of the mangrove root-associated microbial communities. This is most probably linked to the low alpha diversity detected in the endosphere communities (Fig. 1a), which were selectively recruited from the exterior compartment communities.

## Discussion

Understanding the diversity and function of microbial communities along a root-associated fine-scale is crucial in elucidating microbial assembly mechanisms and their ecological importance in mangrove ecosystems. In this study, we analyzed the microbial diversity and their functions across the four root compartments, and their relationships with root exudates. Our results generally support the core hypothesis that the diversity and function of mangrove root-associated microbial communities would diverge along such continuous fine-scale niches. The lower diversity of both bacterial and fungal communities in the endosphere of mangrove root than the diversity of communities in other compartments, was similar to that in the root microbiome of *Arabidopsis*^28^. This points to the selective role of the rhizoplane in controlling microbial entry into the root and reducing microbial diversity^28–30^.

Notably, the microbial function of root-associated microbial communities showed certain patterns in different microenvironments or compartments^2,6,15,31^. In previous studies, plant carbon sources and root exudates were reported to attract microbial populations specifically involved in carbon cycling to the rhizosphere or epsiphere^8,32^. Carbon cycling requires a variety of carbohydrate-active enzymes^33^, including GTs^34^. Some GTs in wheat root environments could help resist pathogenic fungi^35^, whereas other GTs had a self-detoxification mechanism^36^. Also, some GTs could catalyze the activation of hormones in plants and improve the transport efficiency of sugars in roots^34^. In line with these previous findings, our study showed that the abundance of GTs increased from non-rhizosphere, rhizosphere, and episphere to endosphere, suggesting that the internal root microenvironment may have high sugar conversion efficiency and self-detoxification potentials.

Both sediment and rhizosphere of mangrove ecosystems are rich in SRB^10^. As vital decomposers of organic matter in anaerobic environments, SRB play a critical role in the mineralization of organic sulfur and production of available iron and phosphorus for other organisms in mangrove ecosystems^12^. Similarly, we found that root-related compartments had abundant SRB (e.g., *Desulfococcus* and *Desulfosarcina*) and associated functional genes (e.g., *phsA* and *dsrA*). More strikingly, SRB and sulfur reduction-related genes were specifically enriched in the exterior compartments. This is probably due to the presence of oxygen and redox potential gradients in the rhizosphere^37^, which may facilitate sulfate reduction in the non-rhizosphere and rhizosphere soils.

Mangrove ecosystems emit large amounts of methane^3,38^. Previous studies have shown that CH_4_ emissions were remarkably affected by the abundance of methanogens (identified by *mcrA*) and methanotrophs (identified by *pmoA*)^38,39^. In our study, the ratio of *mcrA* to *pmoA* was much higher in the endosphere than in other exterior compartments, indicating that the endosphere had a higher potential of CH_4_ emissions than other compartments. In despite of the previous finding that mangrove sediment has been considered as a key methane sink, this study suggests that CH_4_ production via mangrove roots, and then transport into the above-ground plant may be an alternative but previously unrecognized pathway in mangrove ecosystems, and this phenomenon has been described in previous studies on coastal saltmarsh of *Phragmites*^40^.

Mangrove ecosystems are characterized by rich organic carbon and hypersalinity^5,10^. Under such conditions, ammoniated bacteria have a higher affinity than denitrifying bacteria, enabling dissimilatory nitrate reduction to ammonium (DNRA) to be the main pathway for reserving nitrogen^33,41^. Consistently, genes related to DNRA processes were enriched in root-related compartments in this study. Loaded with the abundant diazotrophic microbes, the three inner compartments of mangrove roots were likely to be rich in ammonia nitrogen. The results indicate the divergence of microbial diversity, abundance, and functions in the four root compartments. Therefore, the endosphere had high sugar conversion efficiency and high potential for self-detoxification, CH_4_ emission, and ammonia nitrogen reserve, while sulfate reduction was stronger in the non-rhizosphere and rhizosphere than in the episphere or endosphere.

Root exudates can affect the microbial community composition in the root environment, especially in the episphere and rhizosphere microenvironments^8^. In previous studies, the role of root exudates was reported to be the recruitment of various functional microbes to protect the host plant^8,9,42^. The high abundance of phenolic acids in root exudates likely results in the high abundance of plant growth-promoting rhizobacteria and their expression of key genes involved in the production of antifungal substances^8,42^. Also, root exudates can increase microbial biomass and diversity by providing a variety of organic matter, including fatty acids and sugars^8^. In this study, we found that root exudates in mangroves include amino acids, organic acids, polyhydroxy acids, sugars, phosphates, polyols, and N-compounds. Among these, phenolic acids (e.g., phthalic acid, salicylic acid) and fatty acids (e.g., palmitic acid, myristic acid, and oleic acid) in high abundances could attract more probiotics that protect host plants^8^. For example, we found that *Hyphomicrobium* (a typical denitrifying bacterium)^43^, *Nitrospirae*, and diazotrophs were positively correlated with the abundant root exudates in the episphere and rhizosphere of mangroves; the presence of these organisms may lead to fast nitrogen cycling in mangrove ecosystems as previously observed^8^.

Deterministic and stochastic assembly occur simultaneously along successional chronosequences and drive the spatial distribution of microbial communities in many ecosystems^44,45^; however, the assembly and selection preferences of root-associated microbial communities remain controversial^15,23^. First, the relative abundance patterns of microbial communities in the root compartments follow certain rules of spatial variation^15^. Similar patterns in rice and *Arabidopsis* microbiomes indicated that the endosphere had a higher proportion of Proteobacteria and Spirochetes than the rhizosphere or non-rhizosphere, whereas Acidobacteria and Chloroflexi were mostly depleted in the endosphere^15,46^. In this study, our results also showed that the abundance of Proteobacteria gradually increased, whereas the abundance of Chloroflexi gradually decreased from the non-rhizosphere to the endosphere. Specifically, we found that SRB such as *Desulfococcus*, *Desulfosarcina*, and *Defluviitalea*, were mostly depleted in the endosphere than in the other three compartments, indicating that the relative abundance of microbial communities among the four root compartments of mangroves followed certain rules from non-rhizosphere to endosphere.

Second, the specific habitat characteristics of mangrove root microenvironments may have important impacts on their microbial community assembly. In previous studies, hydrologic connectivity was reported to be a major factor in structuring microbial communities in most ecosystems^47^ and stochasticity was dominant in the assembly of aquatic environments, including groundwater^48^, bioreactor^49^, and flooded rice paddies^50^. On the contrary, some host-associated bacterial communities, including animal guts^51^, reportedly had a higher proportion of deterministic processes than stochastic processes^52^. In this study, we found that deterministic processes were dominant in the four mangrove root compartments, suggesting that the microbial colonization in mangrove roots was not a passive process and that mangrove plants had a strong selectivity for their associated communities. In line with this notion, Fan et al. observed a decreasing importance of deterministic processes in determining the diazotrophic communities in relation to distance from wheat roots^53^.

Third, many studies indicate that root microbiome acquisition is a continuous process of gradual filtration (two-step or multiple-step selection process)^15,22^, whereas the amplification of microbes in the rhizosphere/episphere prior to the selection of microbes in the rhizosphere/episphere (amplification-selection process) was preferred in other studies^23^. In the present study, many dominant microbial phyla (e.g., Proteobacteria, Spirochetes, Firmicutes, Acidobacteria, and Actinobacteria) were enriched in the episphere to certain degrees; specific phyla (e.g., Proteobacteria, Spirochetes, and Acidobacteria) were selectively enriched in the endosphere. These results revealed that the assembly pattern of mangrove root microbial communities could depend on amplification-selection. To the best of our knowledge, there are no studies wherein assembly processes combined with chronosequence with spatial patterns were conducted in mangrove ecosystems. It is crucial to further explore the factors that influence such assembly processes and underlying mechanisms.

In this study, we showed the spatial and exudation effects of mangrove roots on the diversity, function, and assembly of root-associated microbial communities. Root-associated microbiomes could form four spatially separable compartments and exhibit divergent diversity and function patterns in the mangrove root environment. Also, we found that root exudates played an important roles in the development of root microbiome in the episphere and rhizosphere compartments. In addition, each mangrove root compartment had unique ecological niches for their bacterial and fungal communities. The assembly mechanisms appeared to be represented by the amplification-selection model. This study provides new insights into the understanding of microbial diversity, function, and their assembly mechanisms in the mangrove root environment. Future studies are needed to clarify the mechanisms by which root exudation affects the root microbiome and to explore the microbe-soil-plant interactions in mangrove ecosystems.

## Methods

### Sampling sites, root collection, and environmental properties

The sampling site was located at Shuidong Bay of Maoming City (21°30′38.82″N, 111°0′37.27″E) (Supplementary Fig. 5), Guangdong, China, where natural mangrove communities are dominated by *K. obovata*^26^. Six individual KO saplings were extracted in April 2019, and their root-associated samples were fractionated into four compartments (Supplementary Fig. 6): non-rhizosphere soil (N), rhizosphere soil (R), episphere (P), and endosphere (D). The samples for these compartments were processed as described by Duran et al.^7^ and Edwards et al.^15^. Briefly, roots were collected from mangrove plants, and non-rhizosphere soil was separated from the root by shaking. The rhizosphere soil (~1 mm thickness around the root) that could not be removed by shaking was collected by washing with sterile water. The clean roots were then washed three times to remove the remaining soil and placed into 1× TE buffer supplemented with 0.1% Triton X-100 in a 50 mL Falcon tube. Next, the episphere samples were collected via washing and extensive shaking in 1×TE buffer supplemented with 0.1% Triton X-100. The episphere microbial biomass was collected via filtering the resulting suspension through 0.22 µM pore size membranes (Nuclepore, Whatman, Meterstone, UK). To collect the endosphere microbial biomass, the roots were surface-sterilized for 1 min in 80% ethanol and subsequently sterilized again for 1 min in 0.25% NaClO. All four root compartment samples were stored at −80 °C until DNA extraction. For non-rhizosphere soils, the moisture content, pH, salinity, oxidation reduction potential, ammonium-N, nitrate-N, nitrite-N, total carbon, and total nitrogen were measured as previously described^54^.

### DNA extraction, PCR amplification, and sequencing

Approximately 0.5 g non-rhizosphere and rhizosphere soil with six replicates was used for DNA extraction using a Power Soil DNA Isolation Kit (MoBio, Carlsbad, CA, USA) according to the manufacturer’s instructions with the modified sodium dodecyl sulfate extraction method^55^. The episphere compartment DNA was extracted using a Power Water DNA Isolation Kit (MoBio, Carlsbad, CA, USA) according to the manufacturer’s instructions. DNA from endosphere samples was extracted using a Power Plant DNA Isolation Kit (Mo Bio Laboratories, Inc., Carlsbad, CA, USA) after thorough grinding under liquid nitrogen. The DNA quality based on 260/280 and 260/230 nm ratios was assessed using Nano Drop ND-2000 Spectrophotometer (Thermo Fisher Scientific, MA, USA)^56^. DNA samples with good quality were diluted to 2 ng/μL for subsequent PCR amplification.

The V3−V4 region of 16S rRNA genes and the fungal ITS1 region were amplified using primer pairs (forward primer, 5′-ACTCCTACGGGAGGCAGCA-3′; reverse primer, 5′- GGACTACHVGGG TWTCTAAT-3′^57^; and forward primer, 5′-CTTGGTCATTTAGAGGAAGTAA-3′; reverse primer, 5′-GCTGCGTTCTTCATCGATGC-3′)^58^. PCR amplification was performed in a total volume of 50 μL containing 10 μL buffer, 0.2 μL Q5 high-fidelity DNA polymerase, 10 μL high GC enhancer, 1 μL dNTP, 10 μM of each primer, and 60 ng of microbial community DNA. Thermal cycling conditions were as follows: an initial denaturation at 95 °C for 5 min, followed by 15 cycles at 95 °C for 1 min, 50 °C for 1 min, and 72 °C for 1 min, with a final extension at 72 °C for 7 min. The PCR products from the first step of PCR were purified using VAHTSTM DNA Clean Beads. A second round of PCR was then performed in a 40 μL reaction containing 20 μL 2× Phusion HF MM, 8 μL ddH_2_O, 10 μM of each primer, and 10 μL PCR products from the first step. Thermal cycling conditions were as follows: an initial denaturation at 98 °C for 30 s, followed by 10 cycles at 98 °C for 10 s, 65 °C for 30 s, and 72 °C for 30 s, with a final extension at 72 °C for 5 min. All PCR products were quantified using Quant-iT™ dsDNA HS Reagent and were pooled. High-throughput sequencing of bacterial rRNA genes and fungal ITS genes was performed using Illumina Hiseq 2500 platform (2 × 250 paired ends) at Biomarker Technologies Corporation, Beijing, China.

### Sequence analysis of 16S rRNA and ITS1 gene amplicons

Raw sequences were first processed using Trimmomatic^59^ and FLASH^60^. During filtering, the sequences were trimmed with a moving window of 50-bp and a quality threshold score of 30. The dataset was then simplified by eliminating singletons. Paired 16S rRNA amplicon sequences were then clustered into OTUs by UPARSE^61^ based on a 97% sequence identity using Quantitative Insights into Microbial Ecology^62^ open reference OTU picking strategy with the Greengenes 16S rRNA database (v.13.5) as a reference^63^. The sequences matching “Chloroplast” and “Mitochondria” were excluded from the datasets. ITS sequences were processed using ITSx^64^ and clustered at 97% sequence identity using UPARSE^61^. Fungal OTUs were checked for chimeric sequences using the Uchime reference against a dedicated chimera detection database^65^. We obtained a total of 63,590 and 63,712 high-quality 16S rRNA and ITS gene amplicon sequences per sample, respectively (Supplementary Table 1 and Supplementary Table 2). With >97% sequence identity and removal of low-abundance OTUs (<0.01% of total abundance), the 16S rRNA sequences were clustered into 12,664 OTUs, and ITS sequences into 15,241 OTUs for all samples (Supplementary Table 3).

### Shotgun metagenome sequencing data analysis

One microgram of DNA was used for metagenome sequencing library preparations combined with NEBNext® UltraTM DNA Library Prep Kit for Illumina (NEB, USA) according to the manufacturer’s recommendations. Index codes were added to attribute sequences to each sample; these samples were purified (AMPure XP system), and the libraries were checked using Agilent 2100 Bioanalyzer (Agilent Technologies, CA) and quantified using real-time quantitative PCR. After cluster generation was performed on a cBot Cluster Generation System, paired-end reads (PE150) were performed on an Illumina HiSeq2500 platform. Low-quality (quality score ≤ 38; base *N* > 10 bp; the length of overlap between adapter and reads > 15 bp) paired-end reads were filtered. The metagenomic assembly was performed using Megahit^66^ at default mode. For assembled metagenomes, open reading frames were predicted based on MetaGeneMark v.2.10. A non-redundant gene catalog (Unigenes) was built using CD-HIT v.4.5.8^67^, and quality was controlled with SoapAligner v.2.21. Taxonomic and functional annotation was performed using DIAMOND combined with the NR database (blastp, *e*-value ≤ 1e−5) and KEGG^68^ (http://www.genome.jp/kegg/pathway. html), CAZy^69^ (http://csbl.bmb.uga.edu/dbCAN/), and EggNOG^70^ (http://eggnog.embl.de/version_4.0.beta/,v4.0) databases. The KOs were classified into higher KEGG categories and KEGG pathways. Gene abundances were normalized into TPM counts. Statistics for the mangrove root-associated microbiome metagenome datasets are summarized in Supplementary Table 4.

### Quantitative PCR analysis of 16S rRNA, ITS, and *nifH* gene

qPCR amplification of 16S rRNA genes using the primers 515F and 806R was performed under the following conditions: 98 °C for 2 min, 30 cycles of 98 °C for 30 s, 50 °C for 30 s, and 72 °C for 1 min^71^. ITS1 quantitative PCR amplification was done under the following thermal reaction conditions: 95 °C for 5 min followed by 30 cycles of 95 °C for 30 s, 50 °C for 30 s, and 72 °C for 40 s using the primers ITS1F and ITS2^64^. Both primer pairs for 16S rRNA and ITS1 were the same as those used for their amplicon sequencing. The primer pair *PolF*: 5′-TGCGAYCCSAARGCBGACTC-3′ and *PolR*: 5′-ATSGCCATCATYTCRCCGGA-3′ specific to an approximately 302-bp fragment of *nifH* gene^72^ was used for qPCR amplification under the following conditions: 94 °C for 5 min, 30 cycles of 94 °C for 30 s, 55 °C for 30 s, and 72 °C for 1 min^73^. After amplification, a melting curve was generated by heating the products to 95 °C, cooling them to 65 °C and then gradually heating them to 95 °C at a rate of 0.2 °C/s. The specificity of the amplified products was confirmed according to the melting curve and via gel electrophoresis. Standards with the known copy number of 16S rRNA gene (V3–V4 region), ITS gene (ITS1 region), and *nifH* gene were all serially diluted from 6.03 × 10^9^ to 6.03 × 10^3^ copies/μL; their amplification efficiency was between 95 and 105%.

### Root exudate collection and analysis

Mangrove saplings were carefully transferred to 1-litre hydroponic tubes with sterile Milli-Q water. Hydroponic tubes were incubated at 24 °C for 24 h to collect root exudates, and the resulting solution was immediately extracted with ethyl acetate and dichloromethane in a rotary evaporator and frozen as previously described^74^. Next, the sample was transferred to an Eppendorf (EP) tube and dissolved in 100 μL methanol, and 400 μL extract solution (acetonitrile:methanol = 1:1) containing the internal standard (L-2-Chlorophenylalanine, 2 μg/mL) was added. Afterwards, 100 μL remaining sample was transferred to an EP tube, and 400 μL extract solution (acetonitrile:methanol = 1:1) containing internal standard (L-2-Chlorophenylalanine, 2 μg/mL) was added. After vortexing for 30 s, the sample was sonicated for 10 min in an ice-water bath. Next, the sample was incubated at −40 °C for 1 h and centrifuged at 10,000 × g for 15 min at 4 °C. The 400 μL supernatant was transferred to a sterile tube and dried in a vacuum concentrator at 37 °C. Thereafter, the dried sample was reconstituted in 200 μL 50% acetonitrile via sonication on ice for 10 min. The resulting solution was then centrifuged at 22,542 × *g* for 15 min at 4 °C, and 75 μL supernatant was transferred to sterile glass vial for liquid chromatography–mass spectrometry (LC/MS) analysis.

LC-MS/MS analysis was performed using 1290 Infinity series UHPLC System (Agilent Technologies) equipped with a UPLC BEH Amide column (2.1 × 100 mm, 1.7 μm, Waters). Ion spray voltage floating (ISVF) at 5000 V or −4000 V was applied in the positive or negative modes, respectively. MS raw data files were converted to the mzXML format using ProteoWizard, and processed using the R package XCMS (version 3.2). The data analysis includes peak deconvolution, alignment, and integration processes. The minfrac and cutoff were set as 0.5 and 0.3, respectively. The in-house MS2 database was applied for metabolite identification.

### Statistical analysis

Statistical analyses were performed using the VEGAN package^75^ in R 3.6.0, including alpha-diversity indices (Shannon index and Chao index) and PCoA. The Student’s *t*-test was performed using SPSS and applied to test the significant differences in microbial abundance among compartments. Differentially abundant OTUs were detected using Deseq2 generalized linear model approach^76^.

Source-Tracker analysis, a Bayesian approach, was used to attribute microbial communities in an environmental sink to various potential sources^77,78^. SourceTracker analysis was conducted within an in-house pipeline (http://mem.rcees.ac.cn:8080) which consisted of relevant bioinformatics tools. The percentage value was the statistical average of the Source-Tracker results. For the ecological process analysis^56,79,80^, we calculated the βMNTD (β-mean-nearest taxon distance) using the R function “comdistnt” for the phylogenetic distance between each OTU in one community (*k*) and its closest relative in a second community (*m*). We used βNTI in combination with Bray–Curtis-based Raup–Crick (*RC*_bray_^79^) to quantify the contribution of major ecological processes to the assembly of root-associated microbial communities^56,79,80^. The percentage value was derived from the statistical average of the ecological process results.

### Reporting summary

Further information on research design is available in the Nature Research Reporting Summary linked to this article.

## Supplementary information

Supplementary Information

Reporting Summary

## Data Availability

The nucleotide sequences for amplicon sequencing were deposited in the SRA database under accession numbers PRJNA612579 and PRJNA612600. Metagenomic data were deposited in the SRA database under accession number PRJNA613873.

## References

[1] Thatoi H, Behera BC, Mishra RR, Dutta SK (2012). Biodiversity and biotechnological potential of microorganisms from mangrove ecosystems: a review. Ann. Microbiol..

[2] Liu X (2020). Revealing structure and assembly for rhizophyte-endophyte diazotrophic community in mangrove ecosystem after introduced Sonneratia apetala and Laguncularia racemosa. Sci. Total Environ..

[3] Yu X (2020). Sonneratia apetala introduction alters methane cycling microbial communities and increases methane emissions in mangrove ecosystems. Soil Biol. Biochem..

[4] Alongi DM (2014). Carbon cycling and storage in mangrove forests. Ann. Rev. Mar. Sci..

[5] Srikanth S, Lum SKY, Chen Z (2015). Mangrove root: adaptations and ecological importance. Trees.

[6] Xu J (2018). The structure and function of the global citrus rhizosphere microbiome. Nat. Commun..

[7] Durán P (2018). Microbial interkingdom interactions in roots promote Arabidopsis survival. Cell.

[8] Sasse J, Martinoia E, Northen T (2018). Feed your friends: do plant exudates shape the root microbiome?. Trends Plant Sci..

[9] Bais HP, Weir TL, Perry LG, Gilroy S, Vivanco JM (2006). The role of root exudates in rhizosphere interations with plants and other organisms. Annu. Rev. Plant Biol..

[10] Thatoi H, Behera BC, Mishra RR, Dutta SK (2013). Biodiversity and biotechnological potential of microorganisms from mangrove ecosystems: a review. Ann. Microbiol..

[11] Mckee KL (1993). Soil physicochemical patterns and mangrove species distribution–reciprocal effects?. J. Ecol..

[12] Holguin G, Vazquez P, Bashan Y (2001). The role of sediment microorganisms in the productivity, conservation, and rehabilitation of mangrove ecosystems: an overview. Biol. Fert. Soils.

[13] Reef R, Feller IC, Lovelock CE (2010). Nutrition of mangroves. Tree Physiol..

[14] Xie XY, Weng BS, Cai BP, Dong YR, Yan CL (2014). Effects of arbuscular mycorrhizal inoculation and phosphorus supply on the growth and nutrient uptake of Kandelia obovata (Sheue, Liu & Yong) seedlings in autoclaved soil. Appl. Soil Ecol..

[15] Edwards J (2015). Structure, variation, and assembly of the root-associated microbiomes of rice. Proc. Natl Acad. Sci. USA.

[16] Hartman K, Tringe SG (2019). Interactions between plants and soil shaping the root microbiome under abiotic stress. Biochem. J..

[17] Reinhold-Hurek B, Buenger W, Burbano CS, Sabale M, Hurek T (2015). Roots shaping their microbiome: global hotspots for microbial activity. Annu. Rev. Phytopathol..

[18] Liu YL (2019). Initial utilization of rhizodeposits with rice growth in paddy soils: rhizosphere and N fertilization effects. Geoderma.

[19] Johansson JF, Paul LR, Finlay RD (2004). Microbial interactions in the mycorrhizosphere and their significance for sustainable agriculture. FEMS Microbiol. Ecol..

[20] Ofek-Lalzar M (2014). Niche and host-associated functional signatures of the root surface microbiome. Nat. Commun..

[21] Liu, Y. X., Qin, Y., Chen, T., Lu, M. & Bai, Y. A practical guide to amplicon and metagenomic analysis of microbiome data. *Protein Cell* 1–16 (2020). 10.1007/s13238-020-00724-8 PMC8106563 32394199

[22] Edwards J (2019). Soil domestication by rice cultivation results in plant-soil feedback through shifts in soil microbiota. Genome Biol..

[23] Wang X (2020). An amplification-selection model for quantified rhizosphere microbiota assembly. Sci. Bull..

[24] Vellend BM (2010). Conceptual synthesis in community ecology. Q. Rev. Biol..

[25] Stegen JC, Lin X, Fredrickson JK, Chen X, Konopka A (2013). Quantifying community assembly processes and identifying features that impose them. ISME J..

[26] Peng Y (2016). Virtual increase or latent loss? A reassessment of mangrove populations and their conservation in Guangdong, southern China. Mar. Pollut. Bull..

[27] Zahar FE (2008). Plant host habitat and root exudates shape soil bacterial community structure. ISME J..

[28] Edwards J, Johnson C, Santos-Medellín C, Lurie E, Sundaresan V (2015). Structure, variation, and assembly of the root-associated microbiomes of rice. Proc. Natl Acad. Sci. USA.

[29] Bulgarelli D (2012). Revealing structure and assembly cues for Arabidopsis root-inhabiting bacterial microbiota. Nature.

[30] Lundberg DS (2012). Defining the core Arabidopsis thaliana root microbiome. Nature.

[31] Beckers B, De Beeck MO, Weyens N, Boerjan W, Vangronsveld J (2017). Structural variability and niche differentiation in the rhizosphere and endosphere bacterial microbiome of field-grown poplar trees. Microbiome.

[32] Zhalnina K (2018). Dynamic root exudate chemistry and microbial substrate preferences drive patterns in rhizosphere microbial community assembly. Nat. Microbiol..

[33] Johnston ER (2019). Responses of tundra soil microbial communities to half a decade of experimental warming at two critical depths. Proc. Natl Acad. Sci. USA.

[34] Lairson LL, Henrissat B, Davies GJ, Withers SG (2008). Glycosyltransferases: structures, functions, and mechanisms. Annu. Rev. Biochem..

[35] Gatti M (2019). The Brachypodium distachyon UGT Bradi5gUGT03300 confers type II fusarium head blight resistance in wheat. Plant Pathol..

[36] Gui C (2019). CytA, a reductase in the cytorhodin biosynthesis pathway, inactivates anthracycline drugs in Streptomyces. Commun. Biol..

[37] Uteau D (2015). Oxygen and redox potential gradients in the rhizosphere of alfalfa grown on a loamy soil. J. Plant Nutr. Soil Sci..

[38] He S (2015). Patterns in Wetland microbial community composition and functional gene repertoire associated with methane emissions. Mbio.

[39] Meyer, K. M. et al. Community structure—ecosystem function relationships in the Congo Basin methane cycle depend on the physiological scale of function. *bioRxiv*, 639989 (2019). 10.1111/mec.15442 32285532

[40] van den Berg M, Ingwersen J, Lamers M, Streck T (2016). The role of phragmites in the CH_4_ and CO_2_ fluxes in a minerotrophic peatland in southwest Germany. Biogeosciences.

[41] Dong LF (2011). Dissimilatory reduction of nitrate to ammonium, not denitrification or anammox, dominates benthic nitrate reduction in tropical estuaries. Limnol. Oceanogr..

[42] Ankati S, Rani TS, Podile AR (2019). Changes in root exudates and root proteins in groundnut-Pseudomonas sp. interaction contribute to root colonization by bacteria and defense response of the host. J. Plant Growth Regul..

[43] Kazuya Y, Akiro K, Hideyuki O, Shinnichiro S (2003). Characterization of nitrous oxide reductase from a methylotrophic denitrifying bacterium, *Hyphomicrobium denitrificans* A3151. J. Biochem..

[44] Ofiteru ID (2010). Combined niche and neutral effects in a microbial wastewater treatment community. Proc. Natl Acad. Sci. USA.

[45] Chase JM, Myers JA (2011). Disentangling the importance of ecological niches from stochastic processes across scales. Philos. Trans. R. Soc..

[46] Lundberg DS (2012). Defining the core Arabidopsis thaliana root microbiome. Nature.

[47] Langenheder S, Lindström ES (2019). Factors influencing aquatic and terrestrial bacterial community assembly. Env. Microbiol. Rep..

[48] Zhou J (2014). Stochasticity, succession, and environmental perturbations in a fluidic ecosystem. Proc. Natl Acad. Sci. USA.

[49] Zhou J (2013). Stochastic assembly leads to alternative communities with distinct functions in a bioreactor microbial community. Mbio.

[50] Liu W (2020). Dynamic microbial assembly processes correspond to soil fertility in sustainable paddy agroecosystems. Funct. Ecol..

[51] Mikaelyan A, Thompson CL, Hofer MJ, Brune A (2016). Deterministic assembly of complex bacterial communities in guts of germ-free cockroaches. Appl. Environ. Microb..

[52] Napflin K, Schmid-Hempel P (2018). Host effects on microbiota community assembly. J. Anim. Ecol..

[53] Fan K (2018). Soil pH correlates with the co-occurrence and assemblage process of diazotrophic communities in rhizosphere and bulk soils of wheat fields. Soil Biol. Biochem..

[54] Li M (2019). Population characteristics and influential factors of nitrogen cycling functional genes in heavy metal contaminated soil remediated by biochar and compost. Sci. Total Environ..

[55] Zhou J, Bruns MA, Tiedje JM (1996). DNA recovery from soils of diverse composition. Appl. Environ. Microbiol..

[56] Tu Q (2016). Biogeographic patterns of soil diazotrophic communities across six forests in North America. Mol. Ecol..

[57] Peng, W., Bo, C. & Hua, Z. High throughput sequencing analysis of bacterial communities in soils of a typical Poyang Lake wetland. *Acta Ecol. Sin.* **37**, 1650–1658 (2017).

[58] Blaalid R, Kumar S, Nilsson RH, Abarenkov K, Kauserud H (2013). *ITS1* versus *ITS2* as DNA metabarcodes for fungi. Mol. Ecol. Resour..

[59] Marc L (2012). RobiNA: a user-friendly, integrated software solution for RNA-Seq-based transcriptomics. Nucleic Acids Res..

[60] Tanja M, Salzberg SL (2011). FLASH: fast length adjustment of short reads to improve genome assemblies. Bioinformatics.

[61] Edgar RC (2013). UPARSE: highly accurate OTU sequences from microbial amplicon reads. Nat. Methods.

[62] Caporaso JG (2010). QIIME allows analysis of high-throughput community sequencing data. Nat. Methods.

[63] DeSantis TZ (2006). Greengenes, a chimera-checked 16S rRNA gene database and workbench compatible with ARB. Appl. Environ. Microbiol..

[64] Bengtsson-Palme J (2013). Improved software detection and extraction of *ITS1* and *ITS2* from ribosomal ITS sequences of fungi and other eukaryotes for analysis of environmental sequencing data. Methods Ecol. Evol..

[65] Nilsson RH (2015). A comprehensive, automatically updated fungal ITS sequence dataset for reference-based Chimera control in environmental sequencing efforts. Microbes Environ..

[66] Li D, Liu CM, Luo R, Kunihiko S, Tak-Wah L (2015). MEGAHIT: an ultra-fast single-node solution for large and complex metagenomics assembly via succinct de Bruijn graph. Bioinformatics.

[67] Fu L, Niu B, Zhu Z, Wu S, Li W (2012). CD-HIT: accelerated for clustering the next-generation sequencing data. Bioinformatics.

[68] Yuki M, Masumi I, Shujiro O, Yoshizawa AC, Minoru K (2007). KAAS: an automatic genome annotation and pathway reconstruction server. Nucleic Acids Res..

[69] Eddy SR, Pearson WR (2011). Accelerated profile HMM searches. PLOS Comput. Biol..

[70] Jensen LJ (2008). eggNOG: automated construction and annotation of orthologous groups of genes. Nucleic Acids Res..

[71] Wang P, Chen B, Zhang H (2017). High throughput sequencing analysis of bacterial communities in soils of a typical Poyang Lake wetland. Acta Ecol. Sin..

[72] Franck (2001). Improvement in the RFLP procedure for studying the diversity of *nifH* genes in communities of nitrogen fixers in soil. Res. Microbiol..

[73] Church MJ, Jenkins BD, Karl DM, Zehr JP (2005). Vertical distributions of nitrogen-fixing phylotypes at Stn ALOHA in the oligotrophic North Pacific Ocean. Aquat. Microb. Ecol..

[74] Jin J (2019). Effect of plants and their root exudate on bacterial activities during rhizobacterium-plant remediation of phenol from water. Environ. Int..

[75] Oksanen, J. et al. Package ‘Vegan’. Community Ecology Package, Version 2.0–10. Available at: http://CRAN.R-project.org/package=vegan (2013).

[76] Robinson MD, McCarthy DJ, Smyth GK (2010). edgeR: a Bioconductor package for differential expression analysis of digital gene expression data. Bioinformatics.

[77] Knights D (2011). Bayesian community-wide culture-independent microbial source tracking. Nat. Methods.

[78] Hu Q (2020). Network analysis infers the wilt pathogen invasion associated with non-detrimental bacteria. Npj Biofilms Microbiol..

[79] Stegen JC (2013). Quantifying community assembly processes and identifying features that impose them. ISME J..

[80] Martinez I (2015). The gut microbiota of rural papua new guineans: composition, diversity patterns, and ecological processes. Cell Rep..

