# Supplementary material for: Diversity, function and assembly of mangrove root-associated microbial communities at a continuous fine-scale
Source: NPJ Biofilms Microbiomes. 2020 Nov 12;6:52. doi: 10.1038/s41522-020-00164-6 (PMC7665043; doi:10.1038/s41522-020-00164-6)
Supplement: Supplementary file 1 — Supplementary Information [file 41522_2020_164_MOESM1_ESM.pdf]

SUPPORTING INFORMATION

A. Supplementary tables

Supplementary Table 1. Quality statistics of 16S rRNA gene amplicon sequencing.

Supplementary Table 2. Quality statistics of ITS amplicon sequencing.

Supplementary Table 3. A summary of 16S rRNA and ITS sequence information.

Supplementary Table 4. Summary statistics for mangrove root-associated microbiome metagenome datasets.

B. Supplemental Figures

Supplementary Figure 1. Relative abundance of bacteria at the phylum (a) or genus (c) level of four mangrove root compartments (N: Non-rhizosphere; R: Rhizosphere; P: Episphere; D: Endosphere). (b) depicts the relative abundance of Proteobacteria at the family level, and (d) illustrates the relative abundance of three proteobacterial classes: Alphaproteobacteria (α), Gammaproteobacteria (γ) and Deltaproteobacteria (δ) in four root compartments.

Supplementary Figure 2. Circular maximum likelihood phylogenetic tree of the top 1/100 abundant ITS1 OTUs. Tree leaves on the inner circle highlighted by multiple colors show the affiliation of different phylogenetic clades. The outer circles show the logarithmic value of relative abundances in percentages of each OTU in four root compartments of mangrove *Kandelia obovate*.

Supplementary Figure 3. Heat map of relationships between high abundance bacterial genera and root exudates (*: P <0.001).

Supplementary Figure 4. The ratio of bacteria to fungi among four root compartments were determined by qPCR.

Supplementary Figure 5. Location of the natural mangrove populations. All plants were harvested in spring 2019.

Supplementary Figure 6. Fractionation protocol. For each biological replicate (n = 6), plant individual was dug out and samples were fractionated into non-rhizosphere soil, rhizosphere soil, root episphere and root endosphere compartments.

Supplementary Table 1. Quality statistics of 16S rRNA gene amplicon sequencing.

Sample ID	Compartment	PE Reads	Raw Tags	Clean Tags	GC(%)	Q20(%)	Q30(%)	Effective(%)
KN1	Non-rhizosphere	45,426	42,996	40,181	54.36	97	94.45	88.45
KN2	Non-rhizosphere	65,764	62,972	59,312	55.49	97.41	95.13	90.19
KN3	Non-rhizosphere	56,738	54,373	51,416	55.69	97.43	95.14	90.62
KN4	Non-rhizosphere	47,544	45,911	43,651	55.01	97.41	95.11	91.81
KN5	Non-rhizosphere	55,019	52,868	50,082	55.26	97.36	95.03	91.03
KN6	Non-rhizosphere	69,762	66,695	62,783	55.44	97.2	94.74	90
KR1	Rhizosphere	67,666	64,359	60,509	55.71	97.27	94.88	89.42
KR2	Rhizosphere	56,228	53,513	50,287	56.06	97.22	94.77	89.43
KR3	Rhizosphere	80,017	75,767	70,758	55.76	97.04	94.47	88.43
KR4	Rhizosphere	79,897	75,981	71,349	55.61	97.18	94.73	89.3
KR5	Rhizosphere	79,994	75,867	70,979	55.79	97.22	94.81	88.73
KR6	Rhizosphere	79,947	77,155	73,192	54.96	97.5	95.28	91.55
KP1	Episphere	65,354	62,792	59,630	54.87	97.45	95.2	91.24
KP2	Episphere	63,916	61,533	58,222	54.46	97.52	95.37	91.09
KP3	Episphere	75,900	72,733	68,420	54.44	97.28	94.94	90.14
KP4	Episphere	77,873	74,502	70,062	55	97.36	95.07	89.97
KP5	Episphere	80,029	76,781	72,320	54.8	97.3	94.97	90.37
KP6	Episphere	67,631	65,074	61,431	54.81	97.37	95.07	90.83
KD1	Endosphere	79,920	75,643	71,657	55.31	97.12	94.56	89.66
KD2	Endosphere	80,116	75,983	72,138	55.53	97.11	94.47	90.04
KD3	Endosphere	79,983	75,996	72,508	55.33	97.2	94.66	90.65
KD4	Endosphere	79,754	75,872	72,252	55.45	97.22	94.7	90.59
KD5	Endosphere	79,909	75,299	71,160	54.99	96.98	94.33	89.05
KD6	Endosphere	79,846	75,699	71,862	55.14	97.15	94.63	90

Supplementary Table 2. Quality statistics of ITS amplicon sequencing.

Sample ID	Compartment	PE Reads	Raw Tags	Clean Tags	GC(%)	Q20(%)	Q30(%)	Effective(%)
KN1	Non-rhizosphere	62,498	41,269	40,368	51.23	99.56	98.61	64.59
KN2	Non-rhizosphere	58,307	30,865	30,039	51.86	99.62	98.79	51.52
KN3	Non-rhizosphere	57,228	40,270	38,976	49.42	99.46	98.52	68.11
KN4	Non-rhizosphere	73,793	46,575	45,438	51.78	99.58	98.71	61.57
KN5	Non-rhizosphere	73,339	58,898	57,680	53.81	99.68	98.91	78.65
KN6	Non-rhizosphere	80,065	55,861	54,975	58.73	99.71	98.97	68.66
KR1	Rhizosphere	75,056	68,753	66,019	52.7	99.37	98.23	87.96
KR2	Rhizosphere	79,950	72,943	70,661	54.44	99.44	98.35	88.38
KR3	Rhizosphere	68,902	63,589	60,866	50.25	99.27	98.11	88.34
KR4	Rhizosphere	72,030	65,146	63,531	50.54	99.5	98.47	88.2
KR5	Rhizosphere	80,115	73,619	70,993	48.64	99.51	98.61	88.61
KR6	Rhizosphere	79,977	73,200	70,159	57.13	99.46	98.49	87.72
KP1	Episphere	79,800	74,800	73,350	45.96	99.72	99.04	91.92
KP2	Episphere	79,907	74,948	73,641	45.74	99.77	99.13	92.16
KP3	Episphere	80,200	75,653	74,415	45.94	99.79	99.22	92.79
KP4	Episphere	79,707	74,724	73,141	46.01	99.7	99	91.76
KP5	Episphere	79,877	71,280	68,362	47.61	99.41	98.25	85.58
KP6	Episphere	79,994	71,757	69,223	48.16	99.42	98.3	86.54
KD1	Endosphere	80,126	74,801	71,545	57.09	99.41	98.35	89.29
KD2	Endosphere	80,285	74,293	71,324	56.48	99.38	98.29	88.84
KD3	Endosphere	80,219	75,728	72,579	60.14	99.4	98.35	90.48
KD4	Endosphere	79,938	73,932	71,289	57.35	99.53	98.59	89.18
KD5	Endosphere	79,840	73,431	70,550	60.1	99.46	98.4	88.36
KD6	Endosphere	80,201	73,553	69,960	62.98	99.42	98.3	87.23

Supplementary Table 3. A summary of 16S rRNA and ITS sequence information.

	Total sequence number（Reads）	Total number of high quality sequences（Clean tags）	OTUs	Average high abundance OTUs (> 0.0001)
16S rRNA (V3-V4)	3,341,410	3,003,533	15,489	12,664
ITS (ITS1)	3,618,941	3,064,116	16,034	15,241

Supplementary Table 4. Summary statistics for mangrove root-associated microbiome metagenome datasets.

Sample metagenome	HQ Data (bp)	HQ Reads (%)	HQ Data (%)	GC (%)	Q20 (%)	Q30 (%)
**Non-rhizosphere （N）**	12,465,768,522	99.95	99.53	57.66	98.27	94.78
**Rhizosphere （R）**	12,260,411,716	99.94	99.56	56.41	98.48	95.30
**Episphere（P）**	11,843,466,395	99.94	99.48	57.30	98.01	94.15
**Endosphere （D）**	12,221,404,186	99.92	99.35	53.38	98.08	94.47

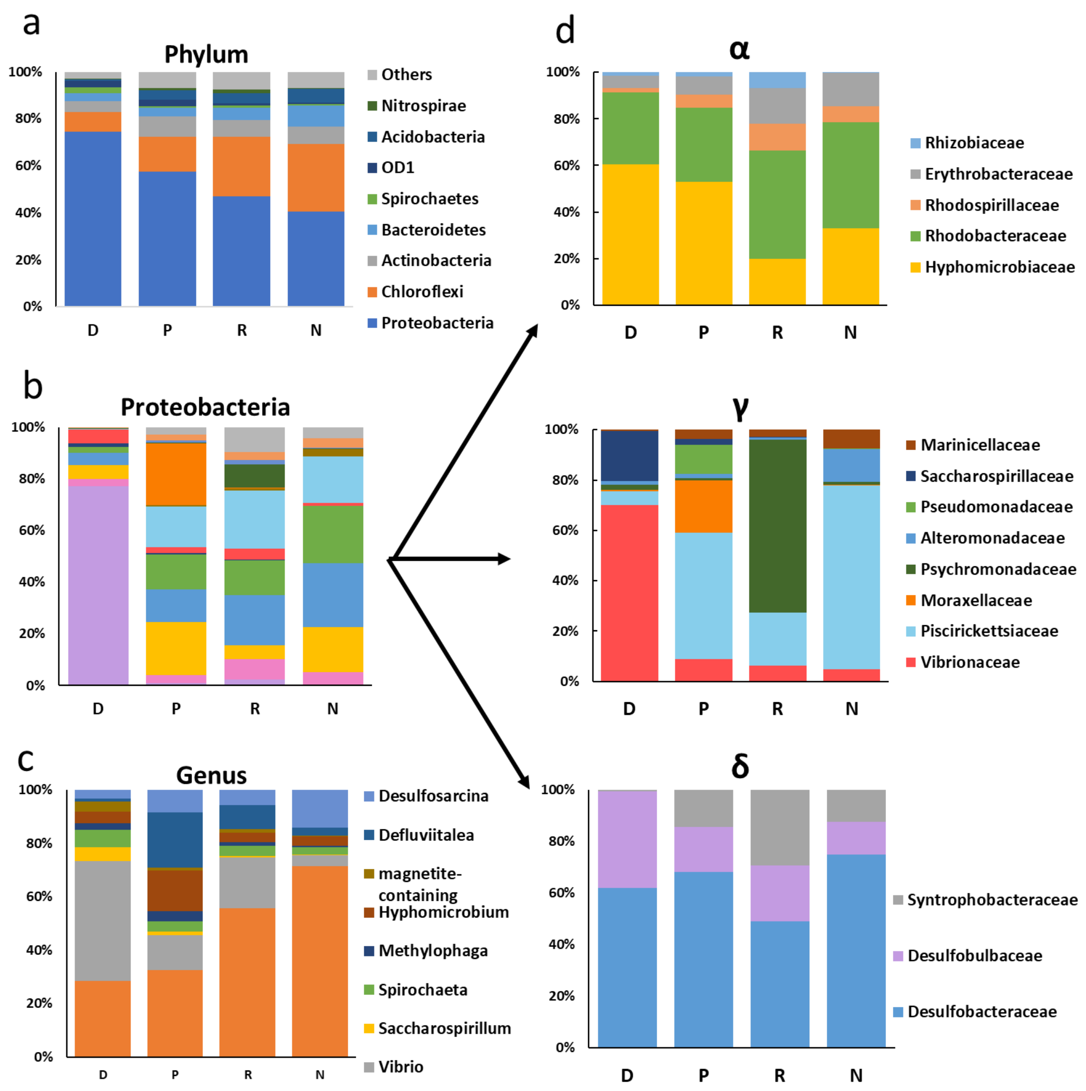

Supplementary Figure 1. Relative abundance of bacteria at the phylum (a) or genus (c) level of four mangrove root-associated compartments (N: Non-rhizosphere; R: Rhizosphere; P: Episphere; D: Endosphere). (b) depicts the relative abundance of Proteobacteria at the family level, and (d) illustrates the relative abundance of three proteobacterial classes: Alphaproteobacteria (α), Gammaproteobacteria (γ) and Deltaproteobacteria (δ) in four root compartments.

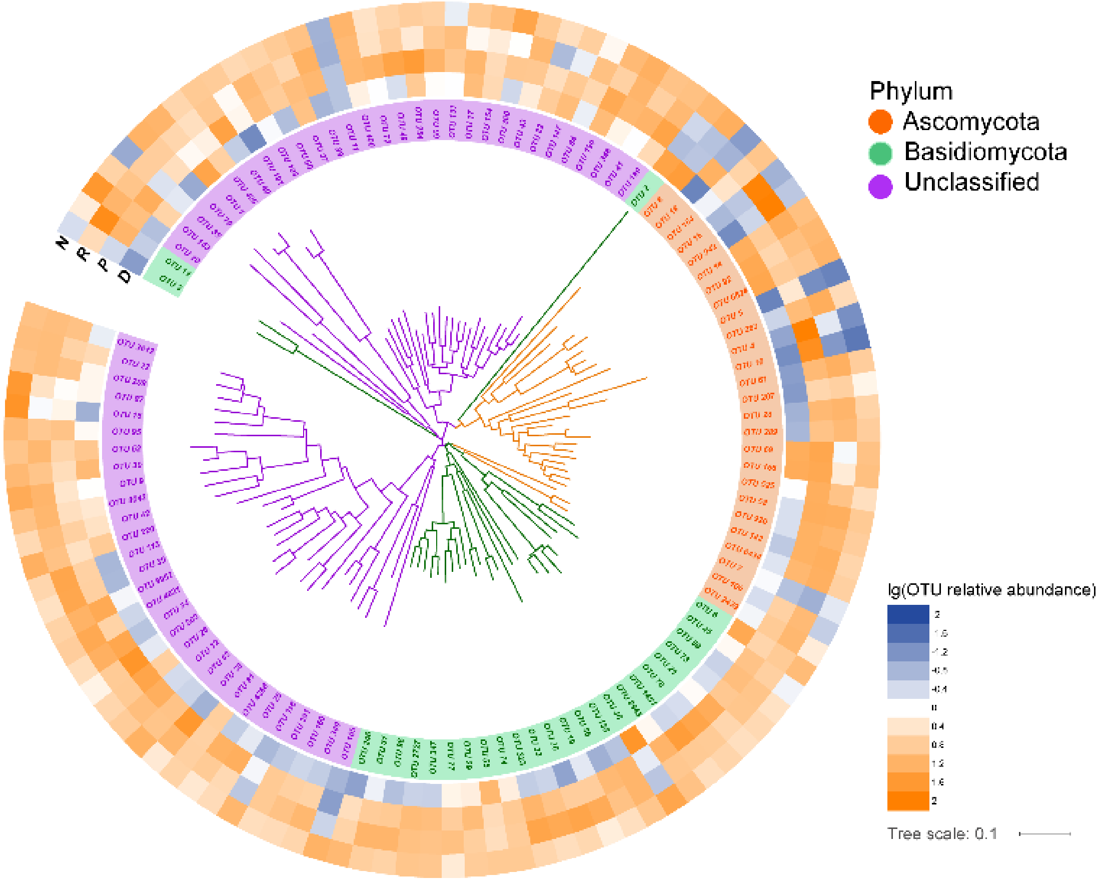

Supplementary Figure 2. Circular maximum likelihood phylogenetic tree of the top 1/100 abundant ITS1 OTUs. Tree leaves on the inner circle highlighted by multiple colors show the affiliation of different phylogenetic clades. The outer circles show the logarithmic value of relative abundances in percentages of each OTU in four root compartments of mangrove *Kandelia obovate*.

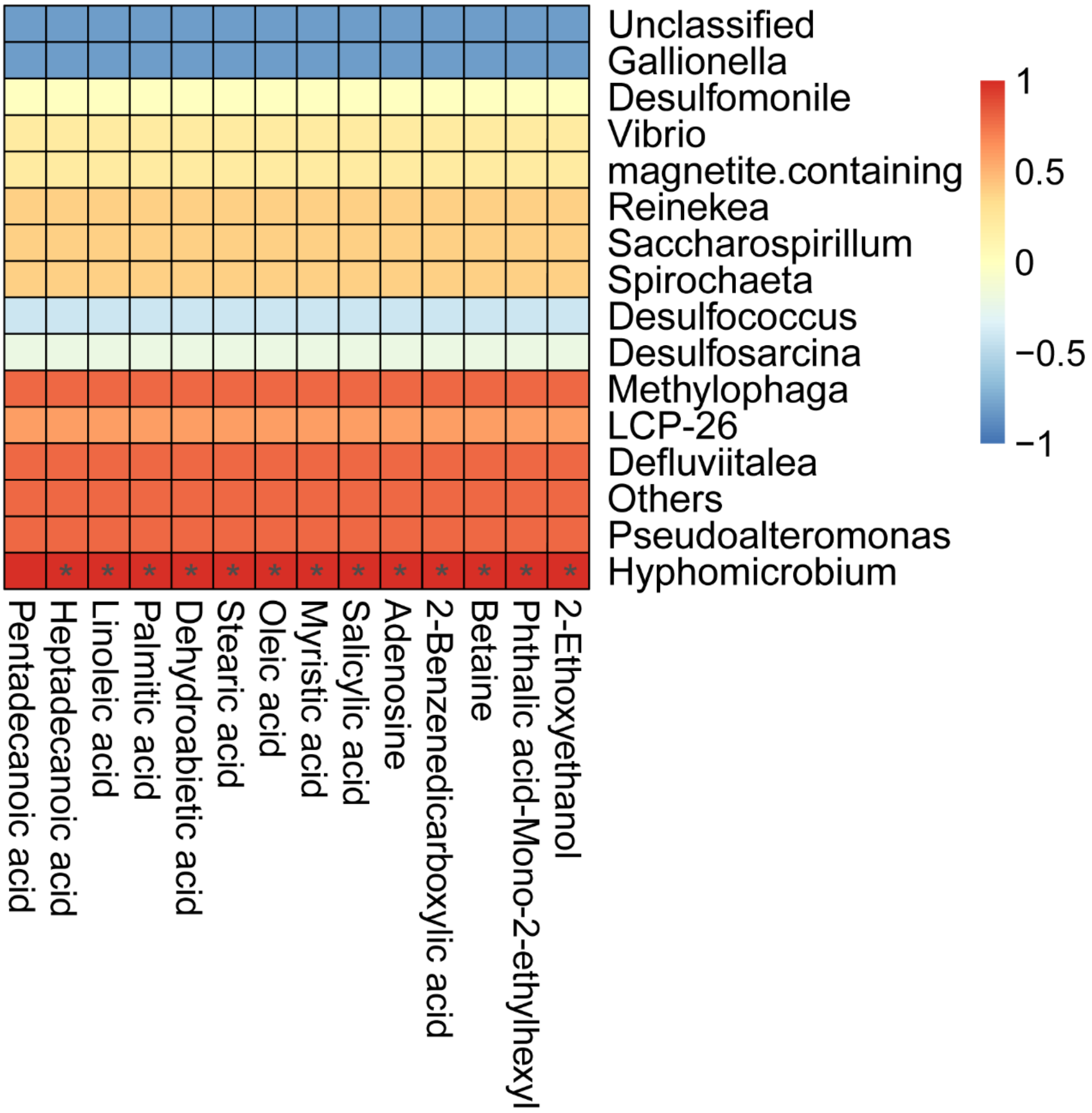

Supplementary Figure 3. Heat map of relationships between high abundance bacterial genera and root exudates (*: $P < 0.001$).

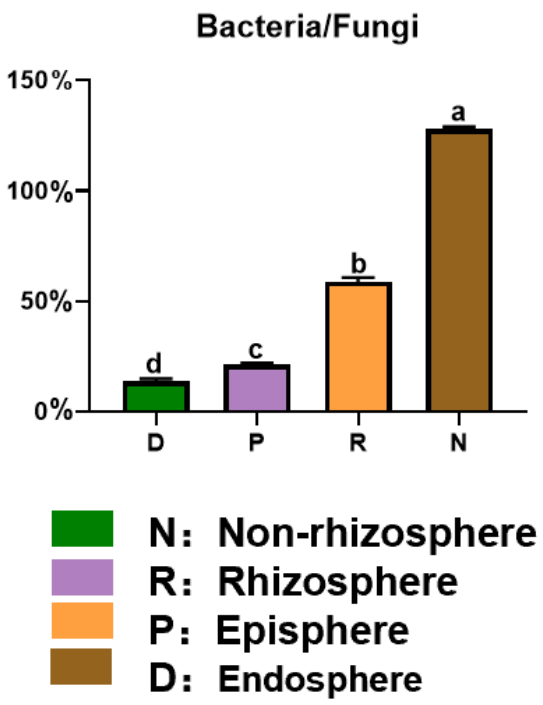

Supplementary Figure 4. The ratio of bacteria to fungi among four root compartments were determined by qPCR.

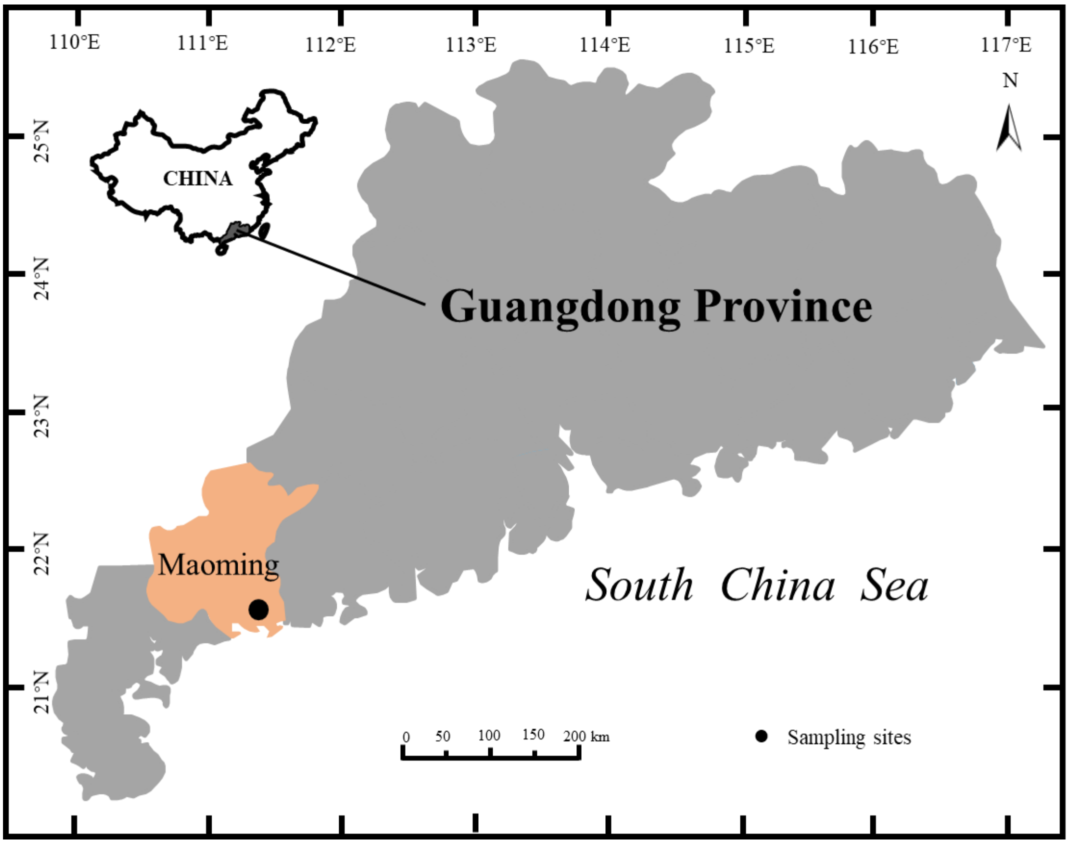

Supplementary Figure 5. Location of the natural mangrove populations. All plants were harvested in spring 2019.

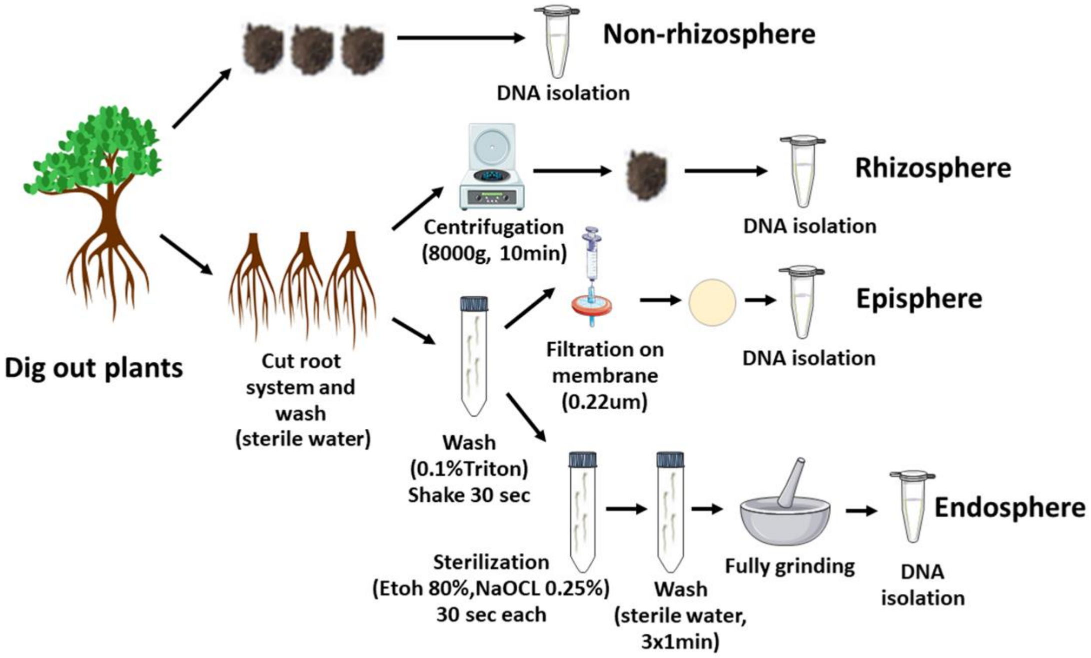

Supplementary Figure 6. Fractionation protocol. For each biological replicate (n = 6), plant individual was dug out and samples were fractionated into non-rhizosphere soil, rhizosphere soil, root episphere and root endosphere compartments.
